# Magnetic Resonance Imaging-Guided Focused Ultrasound Surgery for the Treatment of Symptomatic Uterine Fibroids

**DOI:** 10.1155/2017/2520989

**Published:** 2017-05-03

**Authors:** Laura Geraci, Alessandro Napoli, Carlo Catalano, Massimo Midiri, Cesare Gagliardo

**Affiliations:** ^1^Section of Radiological Sciences, Department of Biopathology and Medical Biotechnologies, University of Palermo, Palermo, Italy; ^2^Radiology Section, Department of Radiological, Oncological and Anatomopathological Sciences, “Sapienza” University of Rome, Rome, Italy

## Abstract

Uterine fibroids, the most common benign tumor in women of childbearing age, may cause symptoms including pelvic pain, menorrhagia, dysmenorrhea, pressure, urinary symptoms, and infertility. Various approaches are available to treat symptomatic uterine fibroids. Magnetic Resonance-guided Focused Ultrasound Surgery (MRgFUS) represents a recently introduced noninvasive safe and effective technique that can be performed without general anesthesia, in an outpatient setting. We review the principles of MRgFUS, describing patient selection criteria for the treatments performed at our center and we present a series of five selected patients with symptomatic uterine fibroids treated with this not yet widely known technique, showing its efficacy in symptom improvement and fibroid volume reduction.

## 1. Introduction

Uterine fibroids (or leiomyomas) are the most common benign tumor of the genital tract in women of reproductive age. According to recent longitudinal studies, the lifetime risk of fibroids in a woman over the age of 45 years is more than 60%, with incidence higher in blacks than in whites [1].

Although they often are asymptomatic, nearly half of women with fibroids have debilitating symptoms, such as menorrhagia, dysmenorrhea, anemia, pelvic pressure or pain, urinary symptoms, constipation, acute pain from degeneration or torsion of a pedunculated fibroid, dyspareunia, infertility, or miscarriage [2].  Table 1 shows a summary of uterine fibroids characteristics.

Several approaches are now available for the treatment of uterine fibroids, including pharmacologic options, such as hormonal therapies and Gonadotropin-Releasing Hormone (GnRH) agonists, and surgical approaches, such as hysterectomy and myomectomy (see Table 2). The new treatment modalities include the following: hysteroscopic resection for submucous fibroids, laparoscopic and vaginal myomectomy, uterine artery embolization (UAE), myolysis by heat, cold coagulation, and laser, laparoscopic uterine artery occlusion, temporary transvaginal uterine artery occlusion, and Magnetic Resonance-guided Focused Ultrasound Surgery (MRgFUS). Factors such as the importance of uterine preservation for patient's desire to become pregnant in the future, symptoms severity, and tumor characteristics may affect the choice of the best possible approach [2, 3].

MRgFUS is a noninvasive thermoablative technique that combines the anatomic detail and thermal monitoring capabilities of Magnetic Resonance Imaging (MRI) with the therapeutic potential of High-Intensity Focused Ultrasound (HI-FU). MRI offers excellent three-dimensional anatomic resolution and real-time thermal monitoring, measuring tissue temperature with an accuracy of ±2°C [4]. HI-FU waves can pass through the anterior abdominal wall and aim at the targeted volume, where tissue temperature increases rapidly up to 60°C or higher, inducing a thermal lesion by protein denaturation and resulting coagulative necrosis, while the skin and overlying tissue layers outside the ablated area remain unaffected [5].

Compared to other treatment options, MRgFUS represents a safe, effective, and noninvasive approach that may be alternatively used as a fertility-preserving technique in selected cases [4].

MRgFUS was approved by the European Community (CE) in 2002 and by the US Food and Drug Administration (FDA) in 2004.

This paper provides an overview of our initial clinical experience with MRgFUS, including a brief description of the treatment system, selection criteria, and procedure workflow, followed by the presentation of a case series of five patients with symptomatic uterine fibroids treated with this technique. These cases have distinctive features that offer good points for discussion.

## 2. Clinical Series

Patients with symptomatic fibroids included in this report were screened by means of a medical examination by a general practitioner or a gynecologist and by a pelvic ultrasounds examination and MRI in order to determine patients' clinical and technical suitability for MRgFUS treatment. We included patient with a definitive diagnosis of uterine fibroid(s) as the cause for their symptoms, uterus size less than 24 cm without the cervix, the presence of less than 6 clinically significant fibroids, absence of contraindications to MRI examination, no massive abdominal scarring that cannot be avoided by manipulations or covered by a US-blocking scar patch (made of a polyethylene film mixed with air bubbles), no evidence of high grade Squamous Intraepithelial Lesion (SIL), and no ongoing pregnancy or unstable medical conditions. All the patients included in this report have read and signed an informed consent which included the use of collected anonymized data for scientific publications.

### 2.1. MRI Selection Criteria

Patients underwent a pelvic MRI screening examination (Signa HDxt 1.5T scanner from GE Medical Systems; Milwaukee, WI, USA) that included the following sequences acquired with 4 mm thick consecutive slices: axial, sagittal, and coronal T2-weighted (T2-w) Fast Recalled Fast Spin Echo (FRFSE), axial T1-weighted (T1-w) Fast Spin Echo (FSE) with and without fat saturation, and, after intravenous (i.v.) administration of 0.1 mmol/kg of gadobenate dimeglumine (MultiHance®, Gd-BOPTA; Bracco Imaging SpA, Milano, Italy), sagittal and coronal T1-w FSE and axial T1-w FSE with fat saturation.

The purpose of the MRI screening examination was to check the accessibility, viability, and texture of the fibroids. MR images were also useful to obtain information on size, location, number, signal intensity on T2-w images, and postcontrast enhancement of uterine fibroid(s) as well as abdominal scars in the intended beam path, presence of adenomyosis, and existence of other uterine disorders or any other pathology outside the uterus. Fibroids suitable for MRgFUS were selected in according with guidelines outlined by Yoon et al. [6], Lénárd et al. [7], and more recently by Mindjuk et al. [8].

**Figure 1:**
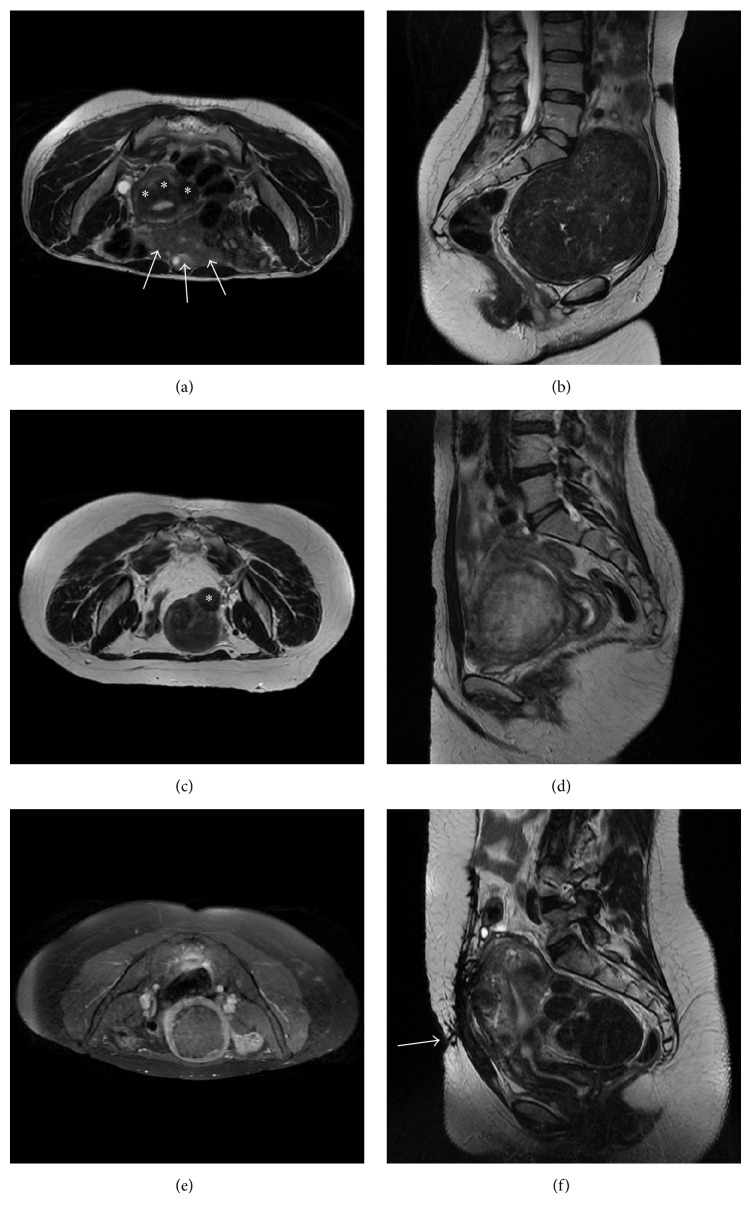
Illustrative pelvic MRI scans of excluded patients: (a) axial T2-w showing three small fibroids (asterisks) and many bowel loops (white arrows) that are interposed between the skin surface and the hypothetic target; (b) sagittal T2-w of a large fibroid which almost occupies the whole pelvis and is dangerously close to sacrum bone and nerves (this patient performed the screening MRI in supine feet first position since she reported some discomfort in maintaining the prone position); (c) axial T2-w showing a small and pedunculated subserosal fibroid (asterisk); (d) sagittal T2-w of a “bright” untreatable cellular fibroid; (e) axial T1-w with fat saturation acquired after intravenous injection of paramagnetic contrast medium showing a nonenhancing fibroid; (f) sagittal T2-w of a patients with a bulky scar in abdominal skin (white arrow).

We included patients with less than 6 clinically significant submucosal, intramural, and/or subserosal fibroids greater than 3 cm (since the smaller sonication spot with our MRgFUS system is 2.5 cm; see asterisks in Figure 1(a)) and smaller than 10 cm (to avoid longer treatment time; Figure 1(b)). Patients with pedunculated fibroids on a small and narrow stalk (less than 50% of fibroid diameter) were excluded as they may potentially disconnect from the uterus after the treatment and require further surgical procedures to remove them from the pelvic cavity (asterisk in Figure 1(c)). Fibroids with a significantly calcified envelope were excluded as well as cellular fibroids showing a bright signal on T2-w images (relative to the uterus wall; see Figure 1(d)) and nonenhancing fibroids on postcontrast T1-w images (Figure 1(e)).

Fibroids with a significant portion (more than 50%) of their volume deeper than 14 cm from the skin line (i.e., the maximum penetration achievable with the HI-FU transducer we used) required mitigation techniques (thinner acoustic coupling gel pad or rectum filling with ultrasound gel) to reduce the distance between the targeted fibroid and the transducer. Special attention has been given to the following elements:

- *Proximity to Sacrum*. The center of the targeted fibroid should be more than 4 cm from any bone surface. Bone can be indirectly heated by the HI-FU far field energy, which could potentially cause secondary nerve heating, resulting in pain and frequent interruptions of the treatment sessions that can result in risk of nerve reversible or permanent injury. Mitigation techniques to avoid sacrum proximity may include HI-FU transducer or beam tilting and rectal filling.
- *Accessibility of the Fibroid(s)*. Bowel (white arrows in Figure 1(a)) and scars (white arrow in Figure 1(f)) may represent an obstacle to the ultrasound beam path. As bowel may contain air or energy absorbing particles, only patients with bowel that can be shifted away from the beam path (by bladder or rectal filling) or by beam angulation may be treated. Extensive abdominal scars may absorb the ultrasound energy and cause pain or result in a skin burn. Therefore, patients with abdominal scars that cannot be avoided using bladder filling and/or beam angulation or that cannot be fully covered by an ultrasound (US) blocking scar patch may not be suitable for MRgFUS.
- *Malignancy*. Patients should not have any imaging finding suggestive of uterine, ovarian, or cervical malignancy on MRI screening evaluation.

### 2.2. Treatment: Planning and Execution

On the day of the treatment, patients are shaved from the umbilicus to the pubis; an i.v. line and a urinary catheter are inserted. The procedure is performed with the patient lying prone (feet toward the MR gantry) on a dedicated MR detachable table which houses the MRgFUS system. In our practice we used an InSightec ExAblate 2100 (InSightec Ltd., Haifa, Israel) MRgFUS system connected to a GE HDxt 1.5T MR scanner (GE Medical Systems; Milwaukee, WI, USA). The abdomen of the patients lies in a water bath of deionized degassed water, in contact with an acoustic coupling gel pad located above the ultrasound transducer.

A light i.v. conscious sedation is administrated by an anesthesiologist to relieve anxiety, prevent movement, and minimize discomfort during the procedure. Patients received 1 gr paracetamol (15 minutes before laying on the MR table) and 0.03 mg/Kg midazolam (at the beginning of the treatment). Additional midazolam boluses (0.01–0.03 mg/Kg) were administered to keep the appropriate sedation level.

As a safety measure, a “stop sonication button” is placed in patient's hand and one more button is available on the workstation that the operating physician uses during treatment. These buttons are capable of the immediate suspension of energy delivery anytime during the procedure.

Preprocedural MRI is essential to evaluate the correct patient positioning and the adequate transducer-to-fibroid alignment. To avoid extensive abdominal scars, bowel, or ovaries, the transducer can be moved and tilted. The HI-FU beam path can be changed by acting on electronic steering through the dedicated software. Moreover, the physician may accordingly modify the position of the uterus by bladder and/or rectal filling (using saline and US gel, resp.).

**Figure 2:**
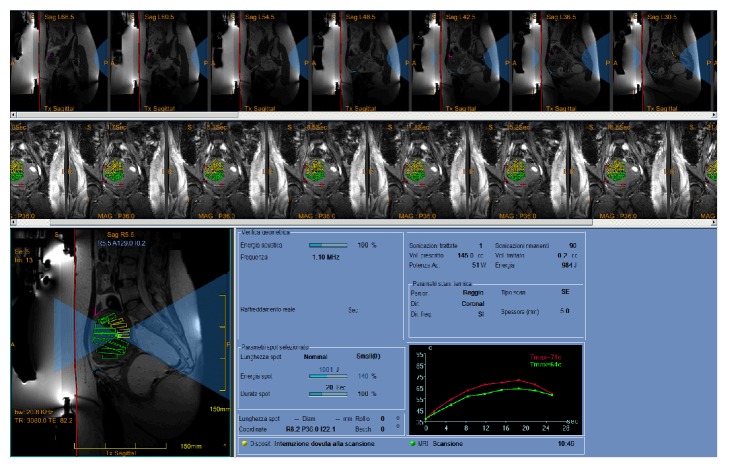
Screenshot from the ExAblate workstation showing the planned target of patient in case 3 after the first sonication performed. In bottom left, the HI-FU beam representation is shown in light blue, the red line indicates the skin-gel pad interface, and critical structures are secured by the use of specific low-energy density region (LEDR) and no-pass regions markers (bowel in pink, pubic bone in light blue). The target volume (region of treatment, ROT) has been split into multiple subvolumes (green and yellow voxels) each of which will be ablated by a specific sonication. Patients' movements during treatment are monitored by reviewing fiducials (red crosshairs) placed by the treating physician on distinct anatomic structures which can be monitored during real-time MR imaging. Real-time thermal mapping after the first sonication is shown in the bottom right graph (maximum temperature achieved in the focal spot is 71°C).

Before starting a treatment, the skin-gel pad interface needs to be defined in the dedicated software (red line in Figure 2). This is followed by marking out the critical structures using specific low-energy density region (LEDR) and no-pass region markers to curtail or prevent the beam path to pass through sensitive organs (see Figure 2: bowel in pink; pubic bone in light blue; sacrum in orange). For added safety, patients' movements during treatment are monitored by reviewing fiducials placed by the treating physician on distinct anatomic structures which can be monitored during real-time MR imaging acquired in treatment (red crosshairs in Figure 2). The target volume, referred to as the region of treatment (ROT), is then defined with its safety margins. A few low-energy sonications (usually up to 3 or 4) are performed for final targeting calibration. Clinical treatment will be performed with multiple therapeutic sonications until a sufficient fibroid volume will be covered (in Figure 2, the ROT has been split into multiple subvolumes each of which will be ablated by a specific sonication). Real-time MR thermometry will reveal any potentially dangerous heating or unwanted exposure. Before the beginning of the treatment, patients are explained what they may feel and if and when they will have to stop the sonication: mild warming, transient pain, and uterine cramping are usually reported by patients during sonications, while acute pain, signs of neurological involvement of the lower limbs, and skin burning sensations require an instantaneous interruption of the energy delivery. Patients are also asked to relay all relevant sensations after each sonication and they are constantly monitored and assessed to check for unusual symptoms.

In our practice, the average treatment time (from the first low-energy sonication to the last therapeutic one) was about 150 minutes.

After the treatment, postcontrast T1-w images are acquired to assess the area of treated tissue (calculated as the nonenhancing area in terms of nonperfused volume, NPV) and to exclude any procedure-related complications.

Even if procedure-related complications are mostly minor and attributable to the effects from focused ultrasound along the whole beam trajectory (near field, targeted site, and far field), the operating physician must be steadily vigilant during the treatment. The most common near field side effect is the risk of skin burns due to bad skin-to-gel pad coupling (scars or skin folds must be carefully avoided and/or settled) or to targeting a site too close to the skin surface. An accurate preparation of the patient (that include proper skin shaving and cleansing and the use of scar patches) usually helps preventing thermal injuries in the near field. In some patients, abdominal wall subcutaneous and muscular edema may occur, but this usually gradually resolves spontaneously [9]. A proper planning and real-time MR imaging prevent target exposures of adjacent organs. Injuries may be also avoided by filling or emptying the bladder via a catheter and/or filling the rectum via a rectal tube for displacing structures such as bowel loops or ovaries out of the HI-FU beam path or to displace the area of treatment with respect to a skin scar; in some cases, the use of bowel mitigation techniques has been also described to treat patients with small fibroids [8]. Far field side effects and risks are attributed to bone and nerves heating due to HI-FU absorbance. During the planning stage of the procedure, these structures are carefully marked (see Figure 2) and, for safety reasons, the minimal distance between the posterior treatment area and bone surface should be no less than 4 cm [10–12]. Potential risk of deep venous thrombosis from lying in a supine position for up to 3-4 hours is substantially mitigated by the use of compression stockings.

We calculated the NPV with an independent workstation (Apple iMac 27′′ late 2013) using the region of interest tool of the Horos software (〈https://www.horosproject.org/〉), a free, open source medical image viewer software based upon OsiriX and other open source medical imaging libraries available under the GNU Lesser General Public License, Version 3 (LGPL-3.0).

An MRI examination follow-up was scheduled at 3, 6, and 12 months after treatment to evaluate the amount of reduction in fibroid volume and to assess the presence of residual viable tissue. The Symptom Severity Score (SSS) was assessed in all the patients who have signed the additional informed consent for research purposes at the time of enrolment and on each MRI follow-up, by the use of the Uterine Fibroids Symptom and Quality of Life (UFS-QoL) questionnaire [13].

We present a selection of five patients with symptomatic uterine fibroids treated with MRgFUS at our center. These cases have distinctive features that offer good points for discussion.

## 3. Case 1

A 43-year-old nulliparous female presented with heavy menstrual bleeding, anemia, pelvic pain, and infertility. The screening MRI showed multiple fibroids (at least six); the three largest ranged from 3.5 cm to 5.7 cm (total volume 107.18 cc) and were hypointense on T2-w images (relative to the uterine wall) and viable on postcontrast T1-w images that were considered clinically significant for patient symptoms (SSS = 61).

In this case, the rectum was filled with about 250 cc of US gel to displace the uterus anteriorly and bowel out of the treatment path. T2-w images were acquired for treatment planning. Three ROT were then defined on the targeted fibroids. Treatment duration was 3 h 55 min (calculated from the first to the last sonication); 102 sonications were performed with an HI-FU energy range of 1600 to 4500 J. The treatment protocol used included small, large, and elongated sonication spots depending on the size of the targeted fibroid. Temperature achieved was in the range of 65–90°C. Contrast-enhanced images were acquired at the end of the treatment and showed a total nonperfused volume (NPV) of 80.45 cc, achieving an NPV ratio (NPV ratio = nonperfused volume/perfused volume expressed as percentage) of 75%.

**Figure 3:**
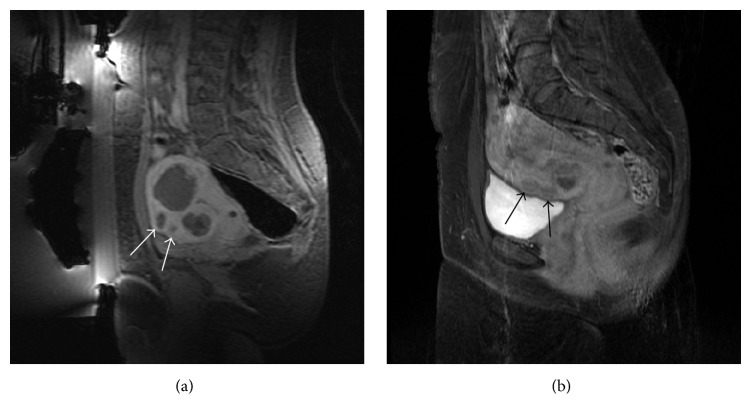
Case 1: (a) sagittal T1-w with fat saturation acquired after intravenous injection of paramagnetic contrast medium showing the two small fibroids (white arrows) located in the near field that were not directly treated but that became nonperfused too after the treatment; two bigger fibroids treated are visible too; (b) a follow-up MRI (T1-w with fat saturation acquired after intravenous injection of paramagnetic contrast medium) showing a normal vascularization of the two small fibroids.

This case illustrates that smaller fibroids located in the near field and not directly treated may show as nonperfused fibroids after treatment (white arrows in Figure 3(a)). A possible hypothesis to explain this finding may lie in damage to feeding vessels shared between targeted fibroid and nontargeted fibroids [14] or, more likely, to a transient vasospasm of the feeding vessels of nontargeted fibroids, since, in a subsequent MRI follow-up, the untargeted fibroids showed back a normal vascularization (black arrows in Figure 3(b)). This finding is not common but should be taken into account when treating patients with similar conditions as it could mislead the physician. At 3, 6, and 12 months of follow-up, her SSS score has reduced to 23, 22, and 18 points, respectively, and the reduction in total tumor size, as a percentage of initial tumor volume, was 42%, 46%, and 50%, respectively.

## 4. Case 2

**Figure 4:**
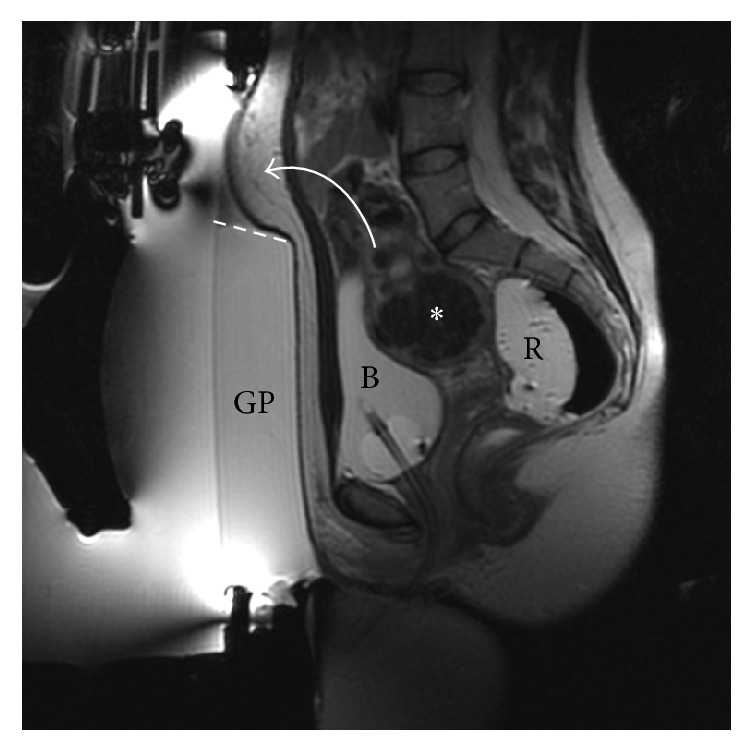
Case 2: sagittal T2-w intraoperative scan showing the targeted fibroid (asterisk) and full bladder (B) with urinary catheter present. Bowels obstructing the beam path were mitigated using a custom sliced (dashed line) gel pad (GP) and rectal filling with ultrasound gel (R). Curved arrow mimics the dislocation of the bowel loops. This treatment was successfully performed through the full bladder.

A 33-year-old female presented with menometrorrhagia and infertility (SSS = 67). The screening MRI showed multiple small fibroids, at least 11, of which there was only one treatable (size: 5 cm; volume: 51.50 cc) intramural fibroid, homogenously hypointense on T2-w images (asterisk in Figure 4), involving the right side of the fundus of the uterus with an heterogeneous enhancement on postcontrast T1-w images. An abdominal scar and some bowel loops were preventing a direct targeting of the region of treatment, but some expedients have allowed us to effectively treat this patient. The abdominal scar was covered by an energy-blocking scar patch to avoid the risk of any skin burns. Bowel obstructing the beam path was mitigated using a custom sliced 45 mm thick cylindrical gel pad (a circular segment equal to about one-sixth of the diameter was sliced off from the cranial part; see dashed line in Figure 4); furthermore, rectum was filled with about 300 cc of US gel and bladder with about 350 cc of saline. Bowel loops were able to be displaced superiorly and out of the beam pathway (curved arrow in Figure 4). The treatment was performed through the full bladder (B in Figure 4) demonstrating how even challenging cases can be treated if the physician succeeds to find a way to expose the ROT. In this patient, we defined only one ROT, around the biggest fibroid, because the other smaller fibroids were not accessible and smaller than 3 cm.

The fibroid was treated in 1 h 40 min, using 43 sonications (energy range: 1100–2250 J; temperature range: 59–102°C). The treatment protocol used included large and elongated spots. Posttreatment, a nonperfused volume of 46 cc and an NPV ratio of 89% were calculated. At 6 months of follow-up, her SSS score has reduced to 35 points and the shrinkage of the treated fibroid was 46% compared to initial fibroid volume.

## 5. Case 3

**Figure 5:**
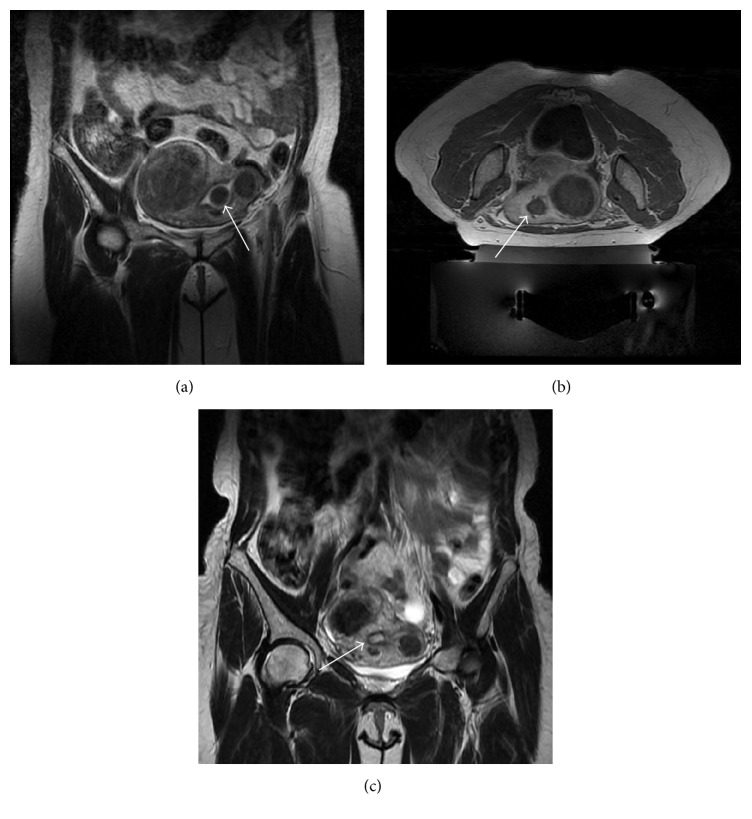
Case 3: (a) screening coronal T2-w showing the bigger intramural fibroid on the right wall of the uterus, the small intracavitary submucosal fibroid on the mid left (white arrow), and one more small intramural fibroid on the left wall of the uterus; (b) axial T1-w acquired after intravenous injection of paramagnetic contrast medium acquired at the end of the treatment showing treated fibroids as nonenhancing round lesions (white arrow on the small intracavitary submucosal fibroid); (c) in a follow-up MRI, the small intracavitary submucosal fibroid was no more appreciable; the white arrow shows the “empty” uterine cavity after spontaneous vaginal expulsion.

A 45-year-old female presented with dysmenorrhea, heavy bleeding, anemia, and pelvic pain (SSS scored 66 points). MRI of the pelvis showed two fibroids, both hypointense on T2-w images; the largest one (diameter 7.3 cm; volume 164.07 cc) was intramural, involving the right side of the fundus of the uterus and the other one was a very small (diameter 2.5 cm; volume 5.73 cc) intracavitary submucosal fibroid (white arrow in Figure 5(a)). Postgadolinium contrast images revealed homogeneous enhancement of both fibroids. Rectum was filled with 250 cc of US gel bringing the uterus closer to the anterior abdominal wall.

Treatment duration was 2 h 15 min, using 82 sonications with an energy range of 1700–5100 J. Temperature achieved was in the range of 60–100°C.

Posttreatment MR images revealed a nonperfused volume of 125.85 cc (NPV ratio of 77%) in the intramural fibroid and a nonperfused volume of 6.01 cc (NPV ratio > 100%) in the intracavitary fibroid.

This case shows that small intracavitary fibroids may be treated safely; ablating an intracavitary fibroid (white arrow in Figure 5(b)) and its stalks will cause its disconnection from uterine wall, followed by spontaneous vaginal expulsion without any complications (see the 3-month follow-up MRI shown in Figure 5(c)).

At 3, 6, and 12 months of follow-up, her SSS score has reduced to 26, 23, and 19 points, respectively, and the shrinkage of the treated intramural fibroid was 40%, 42%, and 49%, respectively, compared to initial fibroid volume.

## 6. Cases 4 and 5

Both 38-year-old (case 4) and 44-year-old (case 5) multiparous females presented with heavy and prolonged menstrual flow, pelvic pain, and pressure. Their SSS were 68 and 65, respectively.

**Figure 6:**
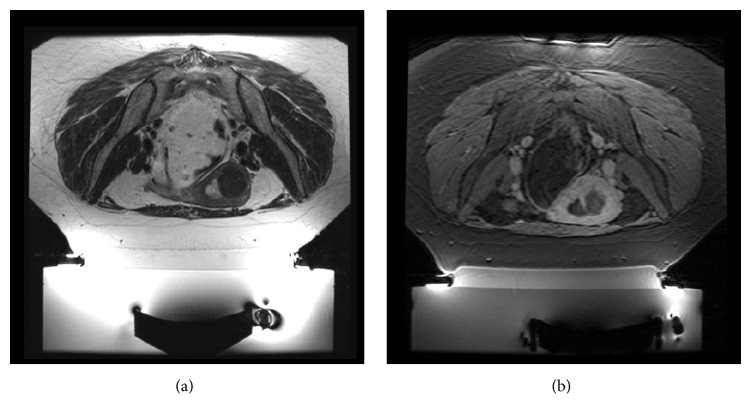
Case 4: example of a treatment with a low ablation rate (NPV = 57%); (a) screening axial T2-w scan; (b) sagittal T1-w with fat saturation acquired after intravenous injection of paramagnetic contrast medium acquired at the end of the treatment.

**Figure 7:**
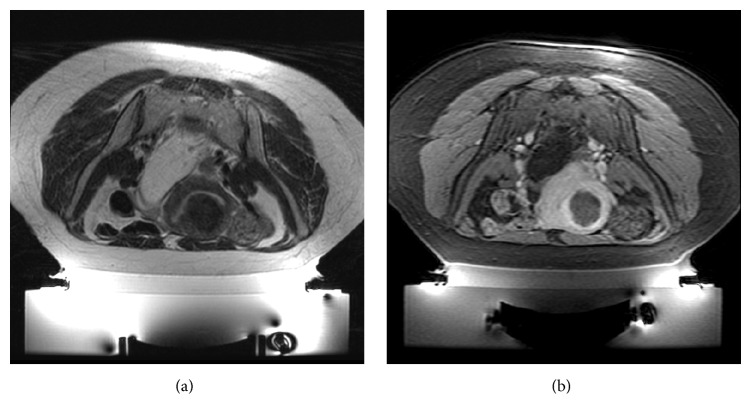
Case 5: example of a treatment with a very high ablation rate (NPV = 99%); (a) screening axial T2-w scan; (b) sagittal T1-w with fat saturation acquired after intravenous injection of paramagnetic contrast medium acquired at the end of the treatment.

The patient in case 4 had a screening MRI demonstrating a single fibroid (3.6 cm in size with volume of 15.19 cc; Figure 6(a)), involving the fundus of the uterus; patient in case 5 showed two fibroids, of which only the larger anterior one (diameter 4 cm; volume 21.45 cc; Figure 7(a)) was treatable because the smaller one was posterior and not fully accessible. In both cases, suitable fibroids were homogenously hypointense on T2-w images and showed homogenous enhancement on postcontrast T1-w images. Patient in case 4 had an abdominal transverse scar that was covered by a scar patch. This patient was treated after filling the rectum with 250 cc of US gel, while patient in case 5 was treated with a partially full bladder to displace bowels superiorly.

Patient in case 4 was treated in 1 h 55 min, using 36 sonications with an energy range of 1259–2770 J using large and elongated spots. Temperature achieved was in the range of 39–96°C. Posttreatment images showed a nonperfused volume of 8.62 cc and NPV ratio of 57% (Figure 6(b)). At 3, 6, and 12 months of follow-up, there was a significant decrease in her SSS score to 21, 19, and 12 points, respectively, and fibroid size had been reduced from baseline by 27%, 62%, and 81%, respectively.

Patient in case 5 was treated in 1 h 15 min, using 31 sonications with an energy range of 1051–446 J using small, large, and elongated spots. Temperature achieved was in the range of 45–97°C. Posttreatment, a nonperfused volume of 21.30 cc and NPV ratio of 99% were achieved (Figure 7(b)). At 3, 6, and 12 months of follow-up, her SSS has reduced to 58, 55, and 52 points, respectively, and the shrinkage of the treated fibroid from baseline was 46%, 55%, and 58%, respectively.

Despite similar symptoms and SSSs, patient in case 4 resulted in only a 57% NPV ratio with a significant decrease in her SSS (from 68 to 21 at the 3-month follow-up) if compared to patient in case 5 that resulted in a 99% NPV ratio with only a slight reduction in her SSS (from 65 to 52 even one year after the treatment). We presented these two cases together to highlight how there is not always a clear correlation between reduction in symptoms and reduction in tumor size even in patients with similar symptoms and SSS.

## 7. Discussion

Several studies have been published focusing on patient symptomatic relief and fibroid shrinkage.

In our experience, all the treatments were successfully completed without complications and the patients were discharged 30 min later after the anesthesiologist confirmed a full recovery from the conscious sedation protocol used. The following day patients returned to their normal daily activities and job outside their home when applicable.

Although this is not always true, in our practice, we have often observed that patients with larger treated volumes have reported an higher degree of symptom improvement and required no alternative treatments. This trend has been already reported in the English literature [15]. Thus, it is fair to always aim at the highest nonperfused volume (at least 60% for a successful treatment), as there is a close relationship between the NPV and clinical outcomes [16]. However, our report suggests that the extent of change in both volume and symptom score varies largely among the patients and there is not always a close correlation between the volume of ablated tissue and the clinical outcome. Nevertheless, there are several predictors of success of an MRgFUS treatment, namely, low signal intensity on T2-weighted images before treatment and nonperfused tissue volume over at least 20% [7].

MRgFUS for uterine fibroids offers several advantages over other fibroid therapies including the following: it is an outpatient procedure, requiring no hospitalization, has a very low complication rate, utilizes no ionizing radiation, and allows the patient to return to her normal daily activities the day after treatment. Furthermore, patients need only conscious sedation allowing patients to communicate with the treating physician and halt treatment if needed.

MRgFUS has a good safety profile. Careful supervision by the operating physician and detailed preparation of the patient are important in minimizing uncommon severe complications as bowel perforation, skin burns, and deep vein thrombosis. It can be carried out even in anemic patients and those who are at risk for surgical operation. It represents an effective and almost noninvasive approach that may be used as a fertility-preserving technique [4]. A number of reports of successful treatment have been published [17–19] showing the feasibility of pregnancy following MRgFUS.

## 8. Conclusion

We have presented a selection of five cases of adult females with symptomatic uterine fibroids treated with MRgFUS. The cases we presented had some distinctive features of interest ranging from technical measures which enabled establishing a safe treatment pathway to uncommon posttreatment findings and demonstration of variable clinical response that does not always correlate with the percentage of tumor ablation calculated. As for all newly introduced therapeutic procedures, many points will be clarified only with further investigations, additional research, and multicenter studies.

MRgFUS represents an effective and almost noninvasive approach that may be used as a fertility-preserving technique for the treatment of symptomatic uterine fibroids. It is a disruptive technology with a remarkable potential in terms of improved outcomes, safety and efficacy, faster recovery, and sustained symptomatic relief and, in the near future, it could cause a sea change in current clinical surgical care. However, this will really happen only when health regulatory systems begin to include this procedure among those reimbursable to extend this treatment option to undeserved patients.

Nowadays, minimally invasive or noninvasive surgical applications are steadily increasing thanks to the continuous technological developments that characterize this particular historical period. Increasingly, imaging-based methods today are used not only for a preliminary assessment or a conventional preoperative planning of many surgical procedures but also for live monitoring too.

MRgFUS is one of the emerging technologies offering treating providers image guidance and thermal monitoring. MRgFUS is a noninvasive safe and effective treatment option for uterine fibroids [20]. The feasibility and the safety of MRgFUS have been tested in a growing number of clinical studies and applications such as painful bone metastases [21], musculoskeletal diseases [22], and various other benign and malignant tumors, like breast cancer [23], bone tumors [24], prostate [25], liver [26], and pancreatic cancer [26, 27] and, more recently, it has been used for noninvasive transcranial applications too [28–32]. MRgFUS is evolving as an alternative or complementary therapy to current treatment options such as surgery, radiotherapy, other thermal ablation procedures, gene therapy, and chemotherapy.

## Figures and Tables

**Table 1:** Summary table for uterine fibroids.

*Etiology*	Unknown

*Incidence*	>60% over the age of 45 years

*Age predilection*	>30 years

*Risk factors*	Age, black race, early age at menarche, familial predisposition, overweight, polycystic ovary syndrome, diabetes, hypertension, nulliparity

*Symptoms*	Menorrhagia, dysmenorrhea, anemia, pelvic pressure or pain, urinary symptoms, constipation, backache or leg pains, dyspareunia, infertility, or miscarriage

*Treatment*	Treatment is required in up to 25% of women. Treatment options include medications, such as gonadotropin-releasing hormone (Gn-RH) agonists, hysterectomy, myomectomy, myolysis, uterine artery embolization, MR-guided Focused Ultrasound Surgery (MRgFUS)

*Prognosis*	Benign tumor, excellent prognosis. In general, they begin to shrink after menopause, and they can grow quickly during pregnancy. They may also bleed into themselves, degenerate, become cystic, calcify, or undergo sarcomatous degeneration (<1% of cases)

*Findings on MR imaging*	Well-defined uterine mass with uniformly low signal intensity as compared to the myometrium on T2-w images and iso-hypointense to the myometrium on T1-w images that enhances homogeneously when gadolinium is administered intravenously. Degenerated fibroids show complex appearance with high or heterogeneous signal on T2-w and postcontrast images

**Table 2:** Comparison of hysterectomy, myomectomy, uterine artery embolization (UAE), and MRgFUS.

Procedure	Hysterectomy	Myomectomy	Uterine artery embolization (UAE)	MRgFUS
Description	Surgical removal of the uterus with or without the cervix. There are several different surgical approaches: vaginal hysterectomy (performed through an incision in the vagina), abdominal hysterectomy (through a horizontal incision on the lower abdomen), and laparoscopic hysterectomy (through four tiny incisions on the abdomen).	Surgical removal of one or more fibroids from within the uterus. It can be performed through several different ways: abdominal myomectomy, laparoscopic myomectomy, and hysteroscopic myomectomy (only for women with submucosal fibroids).	UAE involves blocking, with small particles injected through a catheter, the blood vessels that supply the fibroids, causing them to shrink.	High intensity focused ultrasound waves heat and destroy fibroid tissue. The MRI allows guiding treatment and monitoring tissue temperature in realtime.

Return to normal activities	7 to 56 days.	1 to 44 days.	3 to 10 days,	1 day.

Hospital days	1 to 5 days.	0 to 3 days.	0 to 1 day,	Outpatient procedure; no hospital stay.

Procedure time	1.5 to 3 hours.	1 to 3 hours.	30 minutes to 1.5 hours,	1.5 to 4 hours.

Advantages	Fibroids will not recur because the uterus is removed. The ovaries may be removed or spared.	Only the fibroids are removed; reproductive potential is spared.	Most fibroids can be treated. Incision is small and uterus is retained. Hospital stay is short (1 day) and in some cases may be performed as an outpatient procedure. Recurrence of treated fibroids is very rare. Return to normal activity within 10 days.	Day care procedure requiring no hospitalization, no incisions, no ionizing radiation, no general anesthesia. Severe complications virtually absent. Return to daily activities from the next day of treatment. Fertility is preserved.

Disadvantages/risks	Reproductive potential is lost. Side effects may include early menopause and a reduction in libido. Removal of the ovaries in a premenopausal woman can lead to hot flashes, vaginal dryness, and osteoporosis. Possible surgical risks include bleeding, infections, adhesions, injury to the intestines, or bladder.	Fibroids can regrow and/or new fibroids can develop resulting in recurrent symptoms and additional procedures. The younger the woman is and the more the fibroids are present at the time of myomectomy, the more likely she is to develop fibroids in the future. Possible surgical risks include bleeding, adhesions, and infections.	Low risk of menopause and blockage of blood supply to ovaries. Possible surgical risks include bleeding, uterine infection, blood clots, and injury of the ovaries and to the uterus, potentially leading to a hysterectomy.	Not all type of fibroids can be treated. Fibroids may recur with time. It is a safe procedure with minimal risk; infrequent complications are abdominal pain/cramping, back or leg pain, urinary tract infection, vaginal discharge, skin injury (burns), and transient nerve damage.

Future fertility	Reproductive potential is lost.	Possibility of pregnancy after adequate healing time. A cesarean section may be required for delivery.	Unpredictable effect on fertility.	Fertility is preserved.
